# Genomic representativeness and chimerism in large collections of SAGs and MAGs of marine prokaryoplankton

**DOI:** 10.1186/s40168-024-01848-3

**Published:** 2024-07-15

**Authors:** Tianyi Chang, Gregory S. Gavelis, Julia M. Brown, Ramunas Stepanauskas

**Affiliations:** Bigelow Laboratory for Ocean Sciences, East Boothbay, Maine, 04544 USA

**Keywords:** Single amplified genomes, Metagenome-assembled genomes, Marine prokaryoplankton, Genomic representativeness, Chimerism, Metagenomics

## Abstract

**Background:**

Single amplified genomes (SAGs) and metagenome-assembled genomes (MAGs) are the predominant sources of information about the coding potential of uncultured microbial lineages, but their strengths and limitations remain poorly understood. Here, we performed a direct comparison of two previously published collections of thousands of SAGs and MAGs obtained from the same, global environment.

**Results:**

We found that SAGs were less prone to chimerism and more accurately reflected the relative abundance and the pangenome content of microbial lineages inhabiting the epipelagic of the tropical and subtropical ocean, as compared to MAGs. SAGs were also better suited to link genome information with taxa discovered through 16S rRNA amplicon analyses. Meanwhile, MAGs had the advantage of more readily recovering genomes of rare lineages.

**Conclusions:**

Our analyses revealed the relative strengths and weaknesses of the two most commonly used genome recovery approaches in environmental microbiology. These considerations, as well as the need for better tools for genome quality assessment, should be taken into account when designing studies and interpreting data that involve SAGs or MAGs.

Video Abstract

**Supplementary Information:**

The online version contains supplementary material available at 10.1186/s40168-024-01848-3.

## Introduction

Our current knowledge of the genome content of uncultured microorganisms—which constitute the vast majority of biological diversity on our planet—is derived from two complementary types of data: single amplified genomes (SAGs) and metagenome-assembled genomes (MAGs). Thus far, tens of thousands of SAGs and MAGs have been obtained from the oceans, soils, groundwater, mammalian guts, and other environments [1, 2], substantially expanding our knowledge of microbial diversity, coding potential, evolution, biogeography, biogeochemical roles, and organismal interactions in nature. Furthermore, SAGs and MAGs are increasingly included in reference databases underlying computational tools for microbial taxonomic classification [3, 4] and genome quality control [5], thus contributing to the microbial omics cyberinfrastructure. Therefore, there is a pressing need for a robust understanding of the quality of these relatively new data types, as any errors and biases may propagate and impact a multitude of downstream studies.

Although often assumed to be interchangeable, SAGs and MAGs involve distinct laboratory and computational processes and therefore may be prone to different advantages and limitations. While SAGs are generated by the amplification and sequencing of DNA from individual, physically separated cells, MAGs are products of the computational assembly and binning of shotgun metagenomic reads obtained from a multitude of microorganisms. Several recent publications have reported on the quality of publicly accessible MAGs and, to a lesser extent, SAGs, highlighting genome incompleteness, contamination with sequences from unrelated organisms, and other deficiencies [5–7]. Several studies involving both SAGs and MAGs noted differences in their taxonomic composition [8] and estimated doubling time [9]. However, the comparison of the two methods is not straightforward, due to the broad spectrum of environmental sources and analytical techniques employed to produce the examined datasets. In fact, we are aware of only one prior direct comparison of SAGs and MAGs obtained from the same environment, which included only 16 SAGs and 83 MAGs and therefore may be insufficient to make generalized conclusions [10].

Here, we performed the first systematic comparison of the quality of large sets (thousands of genomes) of SAGs and MAGs from the same environment. SAGs were obtained from the Global Ocean Reference Genomes (GORG) project [1] (specifically GORG Tropics), while MAGs were subsampled from the Ocean Microbiomics Database (OMD) [8]. These recently published datasets represent planktonic prokaryotes from epipelagic seawater samples across various tropical and subtropical regions of the global ocean. Our study unveiled substantial differences between SAGs and MAGs in terms of their taxonomic representativeness, pangenome coverage, and chimerism. These results provide important guidance for improved interpretation and use of SAG and MAG datasets in microbiological research.

## Methods

### Genome sources and quality control

Curated genomes were downloaded from the website https://sunagawalab.ethz.ch for OMD MAGs [8], and the National Center for Biotechnology Information (NCBI) for GORG-Tropics SAGs [1] (BioProject accession PRJEB33281). The previously reported GORG-Tropics SAGs [1] were produced from a randomized set of individual cells obtained from 28 seawater samples of the epipelagic of the Atlantic and Pacific oceans between 40ºN and 40ºS (Additional file 4: Table S1) at Bigelow Laboratory’s Single Cell Genomics Center (SCGC) [11]. The SCGC’s workflow was evaluated for assembly artifacts using three bacterial benchmark cultures with diverse genome complexity and G + C content, indicating no non-target and undefined bases in the assemblies and the following average frequencies of misassemblies, indels, and mismatches per 100 kb: 1.5, 3.0 and 5.0, respectively [11]. The screening of GORG-Tropics SAGs for chimerism included CheckM [6], tetramer frequency analysis [12], and BLAST [13] against the GenBank nr database. The previously reported OMD MAGs [8] were generated from metagenomic reads of individual seawater samples, aided by the abundance correlation profiles produced across a large number of samples, and their quality was assessed by the authors using CheckM, Anvi’o, and dRep [6, 14, 15]. The complete OMD database consists of MAGs, SAGs, and isolate genomes of marine prokaryoplankton from a broad range of geographic locations and depths. To facilitate SAG and MAG comparisons, we retained only OMD MAGs from epipelagic depths and locations that were close to those of the GORG-Tropics SAGs, spanning between ~ 40ºS and 40ºN. MAGs from coastal areas and inland seas, including the Mediterranean Sea, were excluded (Additional file 4: Table S1, Additional file 1: Fig. S1). We named this subset of the OMD dataset “OMD-M”. The GORG-Tropics SAGs and OMD-M MAGs with < 50% CheckM-based completeness estimates were excluded from further analyses, resulting in a similar count of SAGs (4741) and MAGs (4588) (Additional file 5: Table S2). The average, CheckM-based genome completeness estimates for the selected SAGs and MAGs were similar, at 69% and 71%. Two-thirds of the retained MAGs were derived from metagenomes collected and sequenced by Tara Oceans (*n* = 3042) [16], while others originated from BioGEOTRACES [17] expeditions (*n* = 309), the Hawaiian Ocean Time-series (*n* = 460) and the Bermuda-Atlantic Time-series Study (*n* = 777).

### Estimates of taxonomic richness

Taxonomic classification of SAGs and MAGs was performed using GTDB-Tk [4] v1.7.0 with the reference database GTDB-r202 [18]. Taxonomic representativeness of SAGs and MAGs was compared by identifying unique and shared GTDB-Tk-assigned groups at all taxonomic ranks from family to phylum. This approach was not feasible for lower taxonomic levels, due to the limited coverage of marine prokaryoplankton in the GTDB reference database. To generate species-like clusters, SAGs, and MAGs were grouped using dRep [15] v3.2.2 at 95% average nucleotide identity (ANI) (parameters: -sa 0.95 -p 50 -comp 50 -con 10), corresponding to the previously proposed species-level nucleotide identity criterion [19].

### Taxonomic composition comparisons

To avoid potential biases caused by the different sampling methods and atypical environments between the compared datasets, relative abundance analyses were performed only on data derived from 5 to 10 m depth and using MAGs, amplicons, and shotgun metagenome reads recovered from biomass collected on 0.22 μm mesh-size filters following 3 μm mesh-size pre-screens (Additional file 5: Table S2). This resulted in a sub-sample of 4409 SAGs and 1840 MAGs.

In total, 37 16S rDNA-amplicon and 24 shotgun metagenomic datasets (Additional file 4: Table S1) were analyzed, all of which were collected and sequenced by the TARA Oceans team [20]. Low-quality metagenomic reads were removed using Trimmomatic [21] v0.39 with the parameters, LEADING:3 TRAILING:3 SLIDINGWINDOW:4:15 MINLEN:36. For amplicon metagenomic datasets, the taxonomic composition of each dataset was inferred using Kraken2 [22] v2.1.2, which has been demonstrated to achieve high-quality classification on benchmarks [23]. To obtain GTDB-taxonomy, a customized GTDB-r202 database for Kraken2 was constructed using Struo2 [24]. Amplicon sequences classified as “chloroplast”, “mitochondria”, “eukaryote”, and “unclassified” were further removed using the extract_kraken_reads.py script from KrakenTools (https://github.com/jenniferlu717/KrakenTools). For shotgun metagenomic datasets, we applied mOTUs [3] v3.0.1 to infer the taxonomic composition of each dataset. This method classifies and quantifies metagenomic reads based on a set of universal single-copy genes, which were obtained from ~ 600,000 “species”-resolved draft genomes. Therefore, it served as a complementary method of estimating the community structure using the amplicon metagenomic datasets.

For each method (i.e., SAG, MAG, amplicon, and shotgun), the average relative abundances of individual lineages (e.g., phyla and genera) were obtained by calculating the proportion means across samples (e.g., the 24 shotgun metagenomic datasets and 53 SAG samples, see Additional file 4: Table S1). The final relative abundance profile was constructed using the average proportions of individual lineages at different taxonomic ranks (genus-phylum) for each method.

### Gene clustering

Gene-calling in SAGs and MAGs was performed by Prodigal [25] v2.6.3, run in the metagenomic mode (-p meta). For constructing species-like “unigenes” [26] with a minimum ANI threshold of 95%, we used CD-HIT-EST [27] v4.8.1 with the options, -c 0.95 -G 0 -aS 0.9 -g 1 -r 1 -d 0, which followed previous studies [16, 28]. Protein families (PFs) at different AAI thresholds were generated by adjusting the option “–min-seq-id” in MMseqs2 [29] v13.45111, and with the following additional parameter settings following Coelho et al. [26]: (1) -c 0.5 –cov-mode 2 –cluster-mode 0 for PFs grouped at 20%, 30%, and 50% AAIs; and (2) -c 0.9 –cov-mode 1 –cluster-mode 2 for PFs grouped at 90% AAI. Rarefaction analyses were performed using the specaccum function from the R package vegan (https://CRAN.R-project.org/package=vegan) with 1000 rounds of random permutations.

### Fragment recruitment analysis

Post-QC reads from the above-mentioned 24 metagenomic samples were aligned against SAGs and MAGs using BWA-MEM [30] with the minimum sequence overlap and alignment length set to 100 bp.

### Comparison of gene content in *Pelagibacter* and *Prochlorococcus* SAGs, MAGs, and isolates

Since *Pelagibacter* and *Prochlorococcus* represent the most abundant heterotrophic and photosynthetic bacteria, respectively, in tropical surface waters, we performed a deeper analysis on SAGs and MAGs classified to these genera (either “g__Pelagibacter” or “g__Prochlorococcus_A” in GTDB-Tk). For computing 90% AAI PFs and 95% ANI unigene clusters, we used MMseqs2 and CD-HIT-EST with the parameters described above.

For further comparison of gene content, we obtained reference genomes, by searching NCBI Refseq [31] for all the available *Pelagibacter* and *Prochlorococcus* isolate genomes from NCBI Refseq, then used CheckM v1.1.3 to filter for high quality (completeness > 90% and contamination < 5%) genomes for the downstream analyses. This resulted in a selection of 13 *Pelagibacter* and 86 *Prochlorococcus* isolate genomes (Additional file 6: Table S3). To annotate the gene content, we used KofamScan (https://github.com/takaram/kofam_scan) to assign KEGG Orthologs (KO) to putative genes of SAGs, MAGs, and isolates using pre-built prokaryotic HMM profiles, which yielded KEGG modules for the assigned KOs. To minimize erroneous module assignments, we performed the following steps. (1) Anvi'o v7.1 [14] was used to estimate the completeness of KEGG modules in SAGs and MAGs, and a module was considered to be present in a genome if it encompassed at least 75% of the affiliated KOs. (2) we retrieved complete modules assigned to genomes fully annotated in the KEGG GENOME Database (https://www.genome.jp/kegg/genome/). These genomes (KEGG genomes) can be retrieved using organism codes designated by KEGG as follows: for the *Pelagibacter*, “pub”, “phl”, “peg”, and “pel”, and *Prochlorococcus*, “pmb”, “pmc”, “pmf”, “pmg”, “pmh”, “pmj”, “pmm”, “pmn”, “pmt”, “prm”, and “prc”. (3) A module was determined to be present in *Pelagibacter* or *Prochlorococcus* if it occurred in any of the corresponding KEGG genomes or at least 5% of the affiliated SAGs and MAGs (Additional files 7–10: Tables S4–S7). (4) To avoid overestimating the relative gene counts, modules that comprised large fractions of overlapped KOs (≥ 65%, Additional file 11: Table S8) were combined into single representative categories using the R package igraph [32]. (5) To account for the variation in gene counts between KEGG modules, the numbers of putative genes assigned to the module in individual SAGs and MAGs (for both *Pelagibacter* and *Prochlorococcus*) were divided by the median number of assigned genes in individual. (6) Finally, we tested whether these standardized gene counts were significantly different between SAGs and MAGs (for each module), by using a Games-Howell non-parametric post-hoc test (Additional file 12: Table S9 and Additional file 13: Table S10).

### Assessment of chimerism

To estimate the extent of chimeric contamination, SAGs, and MAGs were first analyzed with GUNC [7] v1.0.5 using the proGenomes [33] database v2.1. The flag “–detailed_output” was added to retrieve GUNC's evaluations at all taxonomic ranks. We focused on the following metrics generated by GUNC:

1. Clade Separation Score (CSS) ranges from 0 to 1 and indicates the confidence level of GUNC labeling genomes as chimeras. We applied the default CSS cutoff of 0.45 for calling chimeras, as previously tested and recommended by the developers.
2. Reference Representation Score (RRS) measures the average sequence identity between query genes and references, indicating how well the query genome is represented by the reference genomes.
3. The contamination fraction (“contamination_portion”) shows the proportion of genes failing to be assigned to the major clade for a query genome. We compared the proportions of GUNC-predicted chimeras in SAGs and MAGs using the entire genome datasets as well as genomes that had good representation in GUNC’s reference database (i.e., RRS > 0.5).

Furthermore, SAGs and MAGs were analyzed with MDMcleaner [5] v0.8.2 using default settings except for the specification of the “fast_run” flag.

To identify taxonomic conflicts of 16S rRNA genes in individual SAGs and MAGs, we predicted 16S gene sequences using Barrnap (https://github.com/tseemann/barrnap) v0.9. Taxonomic assignments of 16S gene sequences were performed using a pre-trained QIIME 2 [34] Naive Bayes classifier “Silva 138 99% OTUs full-length sequences” (downloaded from “https://docs.qiime2.org/2023.9/data-resources”), which was trained on the dereplicated full-length 16S gene sequences in the SILVA [35] 138 database.

### Statistical analyses and illustrations

Statistical analyses and figure generation were performed in R v3.6.3 (R Core Team, 2020) using RStudio (v1.3.1093) (RStudio Team, 2020). We used the R package venneuler (https://cran.r-project.org/web/packages/venneuler) for generating Venn diagrams. The R package rstatix (https://cran.r-project.org/web/packages/rstatix) was applied for the Games-Howell and Wilcoxon two-sample paired signed-rank tests.

We used several steps to determine which taxonomic orders had significantly different relative abundances among the analytical methods, as described below. (1) To reduce noise caused by uneven recovery of rare lineages, we first filtered for orders that were prevalent across methods (found in > 90% of the amplicon, shotgun metagenome, SAG, and MAG samples). (2) To reduce false-positive significances caused by low-abundance lineages, we used ALDEx2 [36] to perform Monte Carlo samplings (*n* = 1000) from the Dirichlet distribution on counts of individual orders in each sample (and for each method). The Monte Carlo simulated abundances of individual orders were centered log-ratio transformed to deal with the compositional nature of relative abundance data [37]. (3) Finally, a Games-Howell test was performed using the log-ratio transformed abundances to estimate the abundance-difference of each order between methods and to calculate statistical significance.

To prepare the final illustrations, we used Illustrator (Adobe) as well as the R packages ggplot2 [38], ggpubr (https://cran.r-project.org/web/packages/ggpubr), and RColorBrewer (https://cran.r-project.org/web/packages/RColorBrewer).

## Results

### SAG and MAG taxonomic composition

To evaluate how accurately these two research tools reflect the composition of the studied microbiome, we compared the GTDB-Tk [4]-based taxonomic assignments of the SAGs and MAGs (Additional file 5: Table S2 ) against each other and against data generated using other contemporary techniques: publicly available 16S rRNA amplicons (37 datasets) and shotgun metagenomic reads (24 datasets) of prokaryoplankton inhabiting the tropical and subtropical, epipelagic ocean (Additional file 1: Fig. S1a, Additional file 5: Fig. S2, Additional file 4: Table S1). We found that the relative abundances (Additional file 14: Table S11) of most lineages were not significantly different (significance cutoff: Games-Howell estimated difference > 2; *p* value < 1e − 3, Additional file 15: Table S12) among SAGs, shotgun reads, and amplicons, except for the Marine Group II archaea (Poseidoniales), which had a lower proportion among SAGs (0.88%), as compared to shotgun reads (2.75%) (Games-Howell test: estimated different = 2.42; *p* value = 4e − 7). Meanwhile, many lineages differed by their relative abundances in MAGs, as compared to other datasets. For example, the genus *Pelagibacter* (Pelagibacterales) comprised 36%, 23%, and 15% of SAGs, shotgun reads, and amplicons, respectively, which is comparable to the 24–55% of prokaryoplankton cells identified as Pelagibacterales by fluorescence in situ hybridization in prior studies [39]. However, *Pelagibacter* comprised only 2% of MAGs. Cyanobacteria (dominated by “Prochlorococcus A”) were also underrepresented in MAGs (0.2%), as compared to 7%, 3%, and 3% in SAGs, shotgun reads, and amplicons. Additionally, compared to other techniques, MAGs were overrepresented by genera that constituted a relatively small fraction of other datasets and generally are considered low-abundance taxa in the epipelagic [40, 41], such as members of the Thermoplasmatota and Verrucomicrobiota. A plausible explanation for the lower fraction of Marine Group II archaea among SAGs as compared to shotgun and amplicon reads is the prevalence of polyploidy in Thermoplasmatota [42], which would lead to the overrepresentation of this lineage in community DNA extracts relative to cell counts. These findings suggest that the proportions of microbial lineages in GORG-Tropics SAGs are in general agreement with our current understanding of the composition of marine prokaryoplankton, while substantial taxonomic biases were found in OMD-M MAGs.

**Fig. 1:**
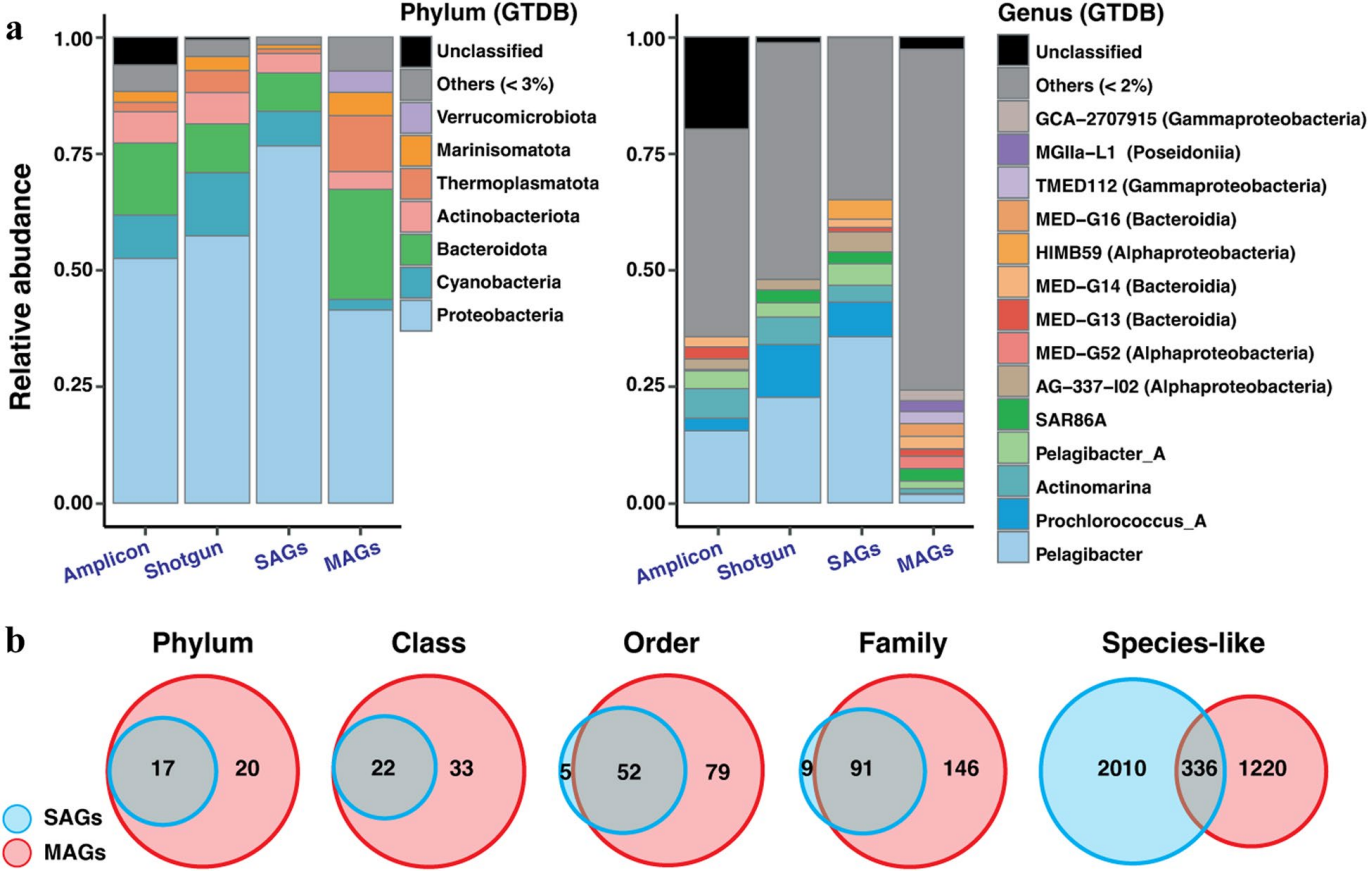
Taxonomic representativeness by SAGs and MAGs. **a** Taxonomic composition of GORG-Tropics SAGs, OMD-M MAGs, 16S rRNA gene amplicons, and shotgun metagenomic reads obtained from prokaryoplankton of the epipelagic of the tropical and subtropical ocean. Lineages constituting < 2% (genus) and 3% (phylum) of either amplicon or shotgun reads were lumped into “Others”. **b** Counts of taxa represented by GORG-Tropics SAGs and OMD-M MAGs

Next, we compared the taxonomic richness of SAGs and MAGs, which revealed a shifting pattern at various taxonomic levels (Fig. 1b). At one extreme, of the 37 phylum-level lineages in the combined SAG and MAG dataset, 20 were represented only by MAGs, and no phyla were found exclusively among SAGs. At another extreme, SAGs represented twice as many unique species-level lineages as compared to MAGs (2010 versus 1220). These findings agree with prior observations [8]. On the one hand, they demonstrate the ability of MAGs, produced from datasets that contain billions of metagenomic reads, to recover genomic information from less abundant taxa that statistically are not expected to be found among a few thousand randomly sampled cells for single-cell genomics. On the other hand, these results indicate that SAGs have a greater capacity than MAGs to resolve prokaryoplankton diversity at a fine phylogenetic resolution.

### Representation of prokaryoplankton genome content

**Fig. 2:**
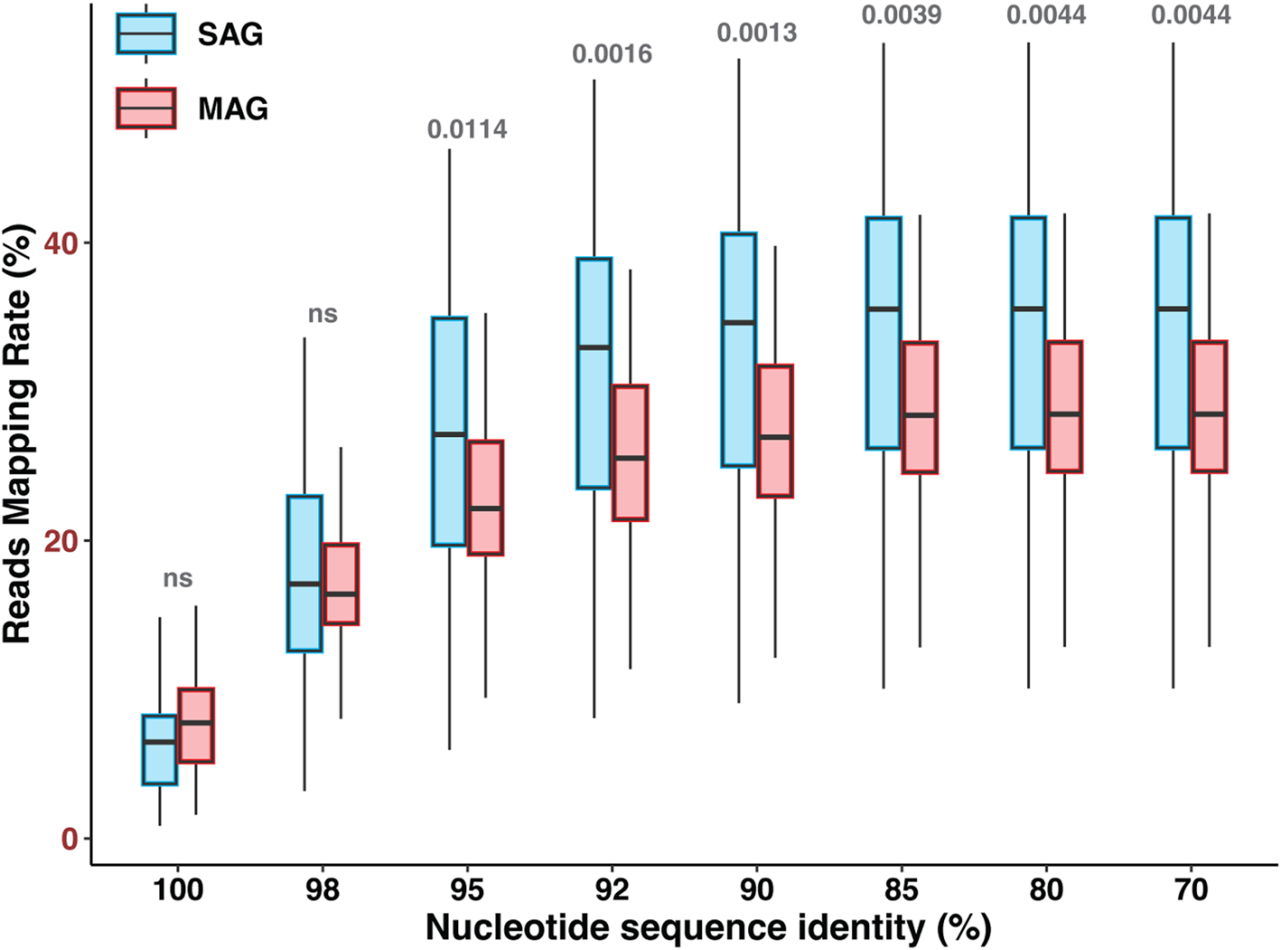
Recruitment of marine metagenome reads against GORG-Tropics SAGs and OMD-M MAGs using various DNA sequence identity thresholds. The statistical significance of SAG and MAG comparisons was determined using the Wilcoxon two-sample paired singed-rank test. *P* values have been shown above each group of boxes, while comparisons with non-significant results are indicated by “ns”

In order to assess the quantitative representativeness of the analyzed SAGs and MAGs, we used them as references to recruit individual reads of the 24 metagenomes—the same datasets that were analyzed in “SAG and MAG taxonomic composition” (Fig. 2). We found no significant differences in read recruitment by SAGs and MAGs when using 100% and 98% nucleotide identity thresholds. Meanwhile, SAGs slightly but significantly outperformed MAGs when using ≤ 95% sequence identity thresholds (Additional file 16: Table S13). This demonstrates that SAGs and MAGs recruit a similar overall fraction of the analyzed metagenomes, although it is important to note the recruitment of reads from various taxa is expected to differ between SAGs and MAGs, due to compositional differences of the two genome datasets (Fig. 1).

**Fig. 3:**
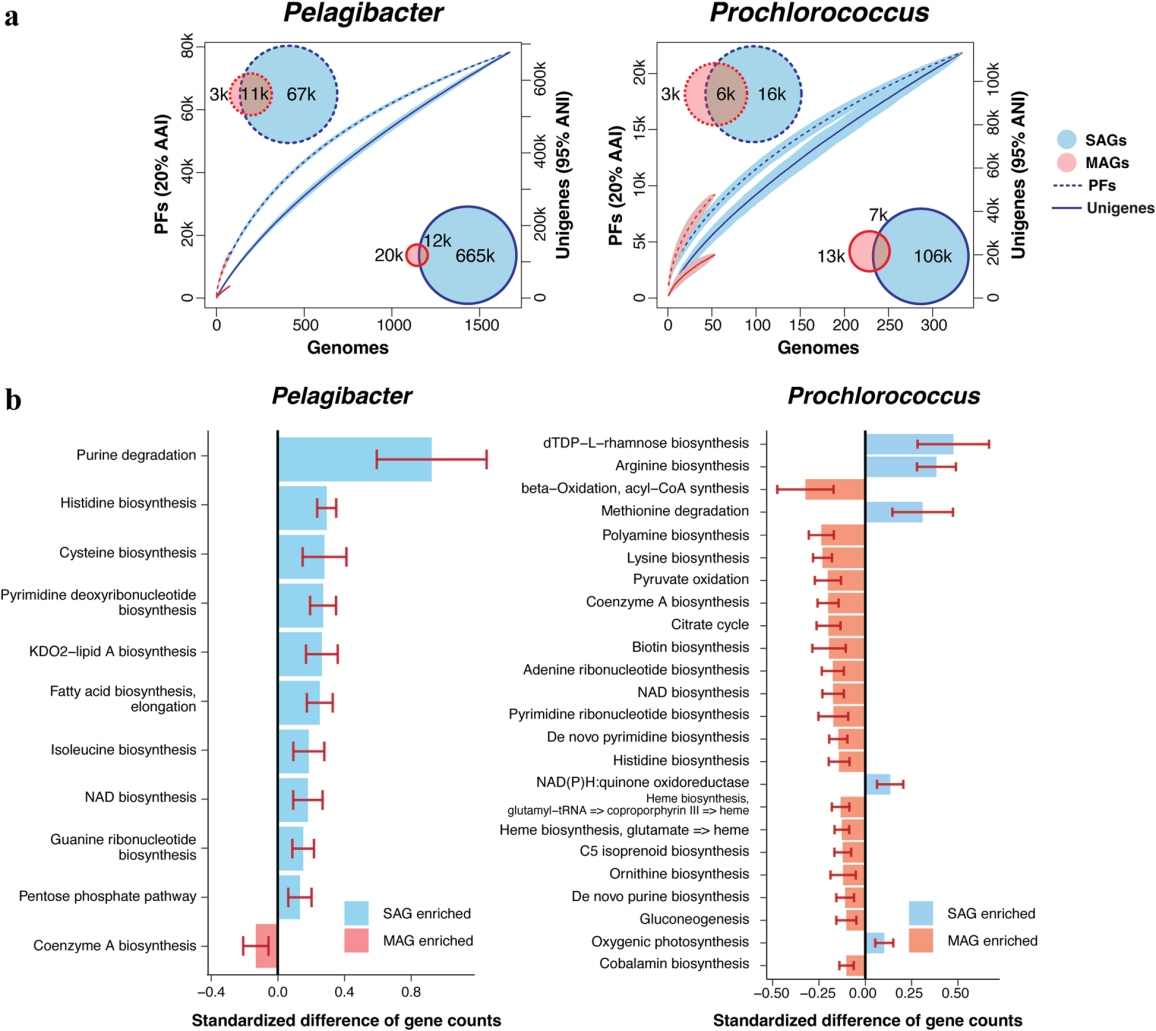
Representation of *Pelagibacter* and *Prochlorococcus* pangenomes by SAGs and MAGs. **a** Comparison of pangenome sizes of *Pelagibacter* and *Prochlorococcus* represented by SAGs and MAGs. The Venn diagrams show the number of shared and exclusive protein families and unigenes encoded by SAGs and MAGs. The rarefaction curves show relationships between the number of genomes and the cumulative count of protein families and unigenes. **b** Gene content differences among *Pelagibacter* and *Prochlorococcus* SAGs and MAGs. For each KEGG module, the standardized difference of gene count is estimated using a Games-Howell nonparametric post-hoc test on gene count differences between individual SAGs and MAGs divided by the median number of genes in individual cultured isolates. Only modules that are significantly (*p* < 1e − 3, Games-Howell test) different between SAGs and MAGs are shown. Bars are color-coded based on whether more genes are estimated to be found in individual SAGs (blue) or MAGs (red) for a given module. Error bars show 95% confidence intervals of the estimated differences derived from the Games-Howell test

Next, we investigated how well SAGs and MAGs represent the pangenomes of *Pelagibacter* and “Prochlorococcus A” (referred to as *Prochlorococcus* from here on)—the most abundant (sub)tropical prokaryoplankton genera with heterotrophic and photosynthetic metabolisms, respectively [43, 44]. This revealed that *Pelagibacter* SAGs encoded 22 × more protein families and 33 × more unigenes than *Pelagibacter* MAGs (Fig. 3a). Likewise, *Prochlorococcus* SAGs encoded 5 × more protein families and 8 × more unigenes than *Prochlorococcus* MAGs. To a large extent, this can be explained by the underrepresentation of *Pelagibacter* and *Prochlorococcus* among MAGs (Fig. 1). Furthermore, a rarefaction analysis revealed that an average SAG of *Pelagibacter* or *Prochlorococcus* contributed more unigenes than an average MAG, while the per-genome contribution of protein families was similar between the two data types. It is noteworthy that neither unigenes nor protein families showed signs of saturation, suggesting that a complete representation of the coding potential of these genera requires further, major scaling up of genome sequencing. Although this analysis is limited to *Pelagibacter* and *Prochlorococcus*, similar patterns are expected from other abundant marine prokaryoplankton lineages, such as HIMB59 (formerly AEGEAN-169), SAR86, Actinomarinales, and others, given their enormous pangenomes [1] and underrepresentation in MAGs (Figs. 1 and 2).

To better understand the specific gene content differences among *Pelagibacter* and *Prochlorococcus* SAGs and MAGs and how they relate to existing cultured isolates, we investigated which KEGG modules have the greatest gene quantity differences between SAGs and MAGs (see Materials and Methods) (Fig. 3b). In the case of *Pelagibacter*, of the 11 modules that differed significantly in relative abundance between SAGs and MAGs, 10 were enriched in SAGs. In contrast, in the case of *Prochlorococcus*, of the 24 modules that differed significantly in relative abundance between SAGs and MAGs, 19 were enriched in MAGs. The KEGG module that was most depleted in *Pelagibacter* MAGs (0.69 versus 1.51 genes per MAG and SAG, on average) participated in the process of purine degradation (Games-Howell test: estimated difference = 0.82; *p* value = 2e − 7) (Additional file 12: Table S9). Genes involved in purine degradation have been found in genomic islands of *Pelagibacter* members in oligotrophic areas and may facilitate the utilization of nucleic acids as nitrogen and carbon sources during starvation [45]. Another *Pelagibacter* module depleted in MAGs is involved in the synthesis of the 3-deoxy-d-manno-octulosonate (KDO) (8.21 versus 11.97 genes per MAG and SAG; estimated difference = 0.23; *p* value = 4e − 7). This eight-carbon acid sugar is an essential component of lipopolysaccharides on the outer membranes of most Gram-negative bacteria and may be important in conveying antiviral and anti-grazing resistance [46]. The patchy distribution of enzymes involved in the biosynthesis of KDO2-lipid A in Proteobacteria also suggests its potential importance in the environmental adaptability of different microbial lineages [47]. In the case of *Prochlorococcus*, genes participating in the synthesis of thymidine diphosphate-L-rhamnose (dTDP-L-rhamnose)—a residue of the O antigen of lipopolysaccharide—were significantly underrepresented in MAGs (0.40 versus 1.58 gens per MAG and SAG; estimated difference = 0.47; *p* value = 6e − 6) (Additional file 13: Table S10). Similar to KDOs, the gain, and loss of dTDP-L-rhamnose synthesis genes in *Prochlorococcus* may be vital to their interactions with viral pathogens, predators, or potential symbionts [48, 49]. Meanwhile, the KEGG modules enriched in MAGs as compared to SAGs included genes involved in the synthesis of Coenzyme A in *Pelagibacter* (3.43 versus 2.96 genes per MAG and SAG; estimated difference = 0.12; *p* value = 9e − 4), and acyl-CoA synthesis (1.04 versus 0.71 genes per MAG and SAG; estimated difference = 0.32; *p* value = 7e − 5) and citrate cycle (8.64 versus 6.85 genes per MAG and SAG; estimated difference = 0.20; *p* value = 4e − 8) in *Prochlorococcus*. This may be explained by the loss of random genomic regions in SAGs, together with the preference for recovering “core” genes in MAGs. These results suggest that MAGs may be depleted in genes considered “flexible”, which often are located on hypervariable genomic islands and have been found to be challenging to MAG assembly and binning algorithms [50–52].

### Genome assembly chimerism

**Fig. 4:**
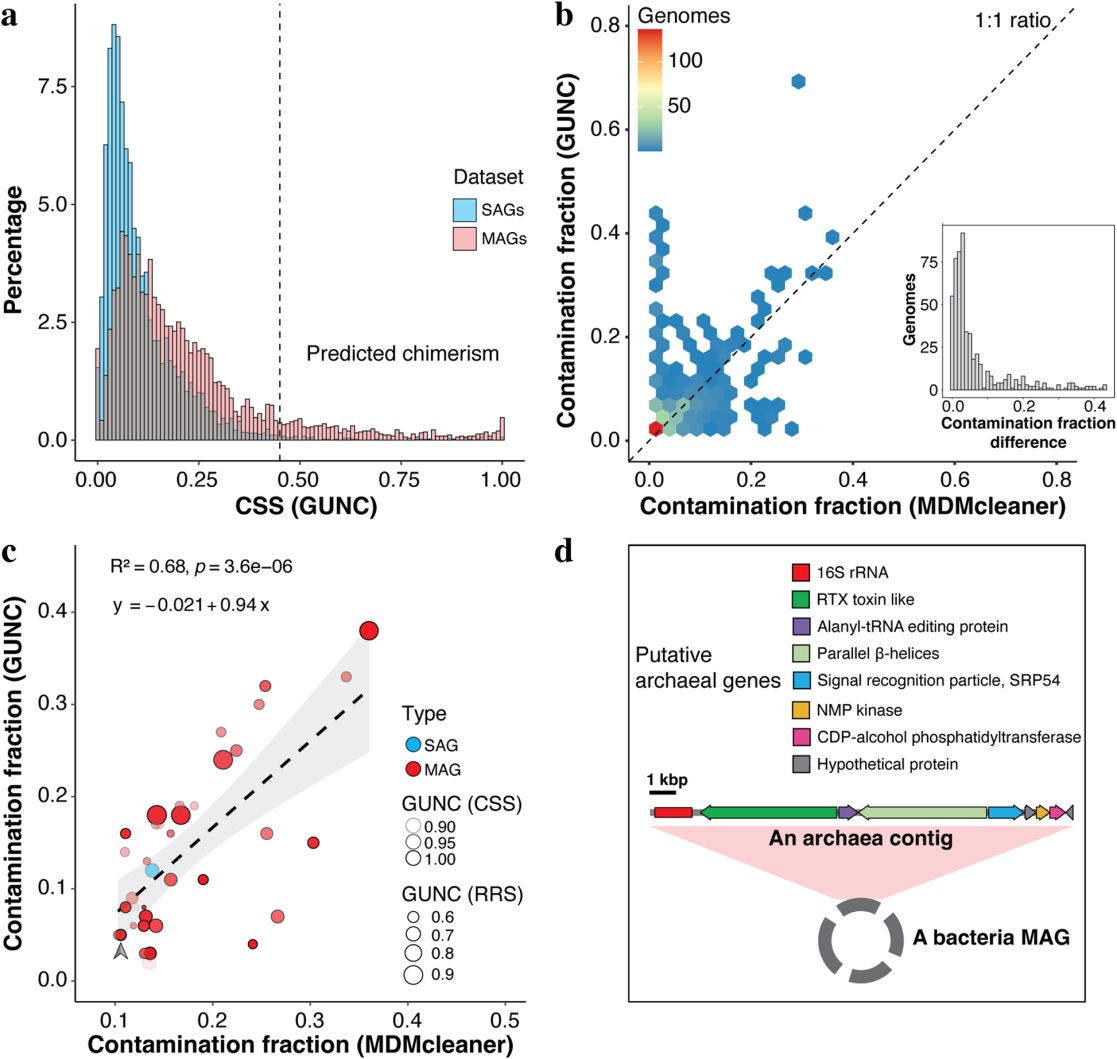
Chimerism in SAGs and MAGs. **a** Distribution of the GUNC clade separation score (CSS) binned at an interval of 0.01. Genomes with CSS > 0.45 are considered chimeric by the default settings of GUNC. Only genomes with a reference representation score (RRS) above 0.5 are shown. **b** Incongruency in contamination fraction estimated by GUNC and MDMcleaner, using the default setting of both tools. The number of genomes in the same space unit is indicated using a color range from blue to red. A 1:1 ratio line is provided as a guide. The histogram shows the distribution of contamination estimate differences between GUNC and MDMcleaner. **c** Contamination fraction estimated by GUNC and MDMcleaner in SAGs and MAGs meeting more stringent criteria (see Materials and Methods). Genomes are represented by individual nodes and are color-coded to indicate whether it is a SAG (blue) or a MAG (red). The transparency and size of nodes indicate values of the GUNC metrics CSS and RRS. Linear regression and its 95% confidence interval are shown as a dashed line and a grey band, and the regression model, *p* value, and coefficient of determination are provided. A specific example shown in panel d has been indicated with an arrow. **d** An example of MAG chimerism, where an archaeal contig (“TARA_SAMEA2622823_METAG-scaffold_5338”), including an archaeal 16S rRNA gene, was included in a MAG (“TARA_SAMEA2622823_METAG_KADMMOJA”) that was classified as a member of the bacterial phylum Marinisomatota by GTDB-Tk

The presence of DNA sequences from multiple taxa in a single genome assembly may lead to false taxonomic classification and misleading inferences about an organism’s coding potential and ecological roles. In this study, we first used the popular computational tool CheckM [6] to detect and filter out GORG-Tropics and OMD-M genome assemblies with > 10% estimated contamination prior to further analyses. This resulted in the removal of 56 MAGs and none of the SAGs (one SAG, AG-901-N13, had a CheckM-contamination estimate of 12%, but was retained, due to the absence of manually verifiable contamination). Prior studies have suggested that CheckM estimates of genome contamination, which are based on the multiplicity of a set of expected single-copy genes, may have limited accuracy [53]. Several new tools for assembly contamination detection, notably GUNC [7] and MDMcleaner [5], were published recently, both reporting a much greater degree of genome chimerism in published datasets, as compared to CheckM results. An implementation of GUNC with default settings indicated potential chimerism in ~ 1% of SAGs and ~ 11% MAGs (Fig. S3). When only considering SAGs and MAGs with good representation in GUNC’s reference database (RSS coefficient > 0.5), estimated chimerism increased to 15% in MAGs, but was unchanged in SAGs (Fig. 4a). However, we found a remarkable degree of incongruence between GUNC and MDMcleaner when using their default settings: some genomes were deemed up to 43% contaminated by one method while found contamination-free by the other (Fig. 4b and Additional file 5: Table S2). We assumed that the limited coverage of marine prokaryoplankton by GUNC and MDMcleaner reference genome databases contributed to this discrepancy, and further considered only cases of chimerism when the following conditions were met: (a) GUNC RRS ≥ 0.5 (this coefficient measures genome’s representativeness in reference database); (b) GUNC CSS ≥ 0.85 (this coefficient indicates GUNC confidence in a chimera call); (c) GUNC contamination detected above the genus level; and (d) MDMcleaner-estimated contamination fraction ≥ 10%. Contamination estimates passing these filters were more consistent between GUNC and MDMcleaner, although differences between these two tools and CheckM (all analyzed SAGs and MAGs had < 10% CheckM-based contamination) were still substantial (Fig. 4c). Cases of genome chimerism conforming to these criteria included one GORG-Tropics SAG (“AG-410-O08”) and 36 OMD-M MAGs (Additional file 17: Table S14). Our further manual inspection confirmed that these 37 assemblies indeed contain sequences derived from taxonomically divergent organisms. As an example, one of the MAGs was dominated by bacterial sequences and was placed within the phylum Marinisomatota by GTDB-Tk, yet contained a 16-kbp contig with close homology to archaeal genomes, and encoded an archaeal 16S rRNA gene affiliated to the phylum Thermoplasmatota (> 98% ANI) (Fig. 4d). These findings indicate a substantially higher frequency of chimerism among OMD-M MAGs, as compared to GORG-Tropics SAGs. They also demonstrate major discrepancies among CheckM, GUNC, and MDMcleaner, indicating that further improvements in the reliability of genome chimerism detection tools are needed.

The 16S rRNA gene has been by far the most broadly utilized phylogenetic marker in microbiology since the 1970s [54]. 16S gene sequences were found in 3137 (66%) SAGs and 368 (8%) MAGs, demonstrating a substantially greater capacity of SAGs to recover this important phylogenetic marker and link it to genome content information (Additional file 5: Table S2). When comparing the genome-based GTDB-Tk and 16S RNA-based QIIME 2 taxonomic assignments, we found taxonomic domain-level mismatches in 0 SAGs and 4 (0.1%) MAGs, phylum-level mismatches in 4 (0.1%) SAGs and 20 (0.4%) MAGs, and class-level mismatches in 7 (0.1%) SAGs and 34 (0.7%) MAGs. Some of these mismatches may be caused by differences in the taxonomic systems used by GTDB-Tk and QIIME 2 and by potential performance limitations of these tools, especially when analyzing taxa with limited representation in underlying databases. Furthermore, mismatches at phylum and lower taxonomic levels may be underestimated, due to the inability of GTDB-Tk and QIIME to assign taxonomies to some of the SAGs and MAGs. However, binning errors are the most plausible explanation for the presence of domain-level classification mismatches in MAGs and the elevated frequency of mismatches at lower taxonomic levels in MAGs as compared to SAGs. The presence of multiple 16S gene copies with conflicting taxonomic assignments at domain (MAG TARA_SAMEA2622823_METAG_KADMMOJA) and phylum levels (MAG BATS_SAMN07137072_METAG_DMGBPHJB), with no such cases detected in SAGs, provides further evidence for binning errors in MAGs when handling contigs containing 16S rRNA genes. Difficulties in the accurate binning of rRNA operons into MAGs are well known and stem from their divergent k-mer composition, as compared to protein-coding genome regions [5]. Overall, this analysis suggests a substantially higher frequency and accuracy of the recovery of 16S rRNA ribosomal genes in SAGs, as compared to MAGs.

## Discussion

The comparison of large sets of SAGs and MAGs obtained from the same environment revealed major differences between the two methods. We found that a microbiome’s taxonomic composition could be reliably analyzed using direct counts of SAGs but not MAGs (Fig. 1). Prior to GORG-Tropics, most SAG collections were relatively small and involved a selective process based on taxonomic or other criteria, thus biasing their composition relative to the original microbiome [12, 55]. Intentionally non-random sets of SAGs are also often produced by cell sorting with probes that target either specific taxa or cells expressing particular phenotypes [56]. Both MAGs and non-randomized SAGs can still be instrumental in quantitative microbiome compositional analyses indirectly when used as references in the recruitment and classification of unassembled metagenomic reads [12, 57]. Here, we provide some of the first evidence that large, randomized sets of SAGs, such as GORG-Tropics, may offer a quantitatively accurate representation of the taxonomic composition of complex microbial communities, complementing environmental microbiologists’ toolkit. Currently, relatively high costs of SAG generation and sequencing limit their broader application. However, as higher-throughput and cheaper technologies emerge [58], we expect SAGs to be increasingly utilized at a scale sufficient to adequately represent complex microbiomes while maintaining the maximal single-cell resolution of biological information.

Biases in the taxonomic composition of MAGs are not surprising, given that each MAG is expected to represent a consensus assembly of a single, near-clonal population, independent of that population’s relative abundance. Thus, methods for MAG assembly and binning were not designed for and should not be used in the quantification of microbial lineages. However, this is not always taken into consideration by microbial ecologists, who sometimes use MAG composition as a metric of absolute [59] or relative [60] taxonomic abundance of microbial taxa in analyzed environments. Our results indicate that such use of MAGs may lead to flawed conclusions. One benefit of the non-proportional composition of MAGs is that they are not dominated by the most abundant taxa, leading to a broader taxonomic spectrum represented in the same number of MAGs, as compared to randomized SAGs.

A substantial limitation of both SAGs and MAGs is their incomplete and variable genome recovery. In the case of SAGs, this is primarily caused by the stochasticity of whole genome amplification when the starting template consists of one or a few DNA molecules, resulting in the under-amplification or loss of random genomic regions [61, 62]. Several techniques have recently been proposed to improve single-cell DNA amplification [11, 62]. The causes of MAG incompleteness are less understood. However, the underrepresentation of hypervariable regions in MAGs, indicated by this (Fig. 3) and prior studies [63, 64] suggests that genomic differences among cells represented by individual MAGs are a contributing factor. Accordingly, the original report of GORG-Tropics found no two identical genomes, even among 6236 randomly picked cells from a 0.4-mL seawater sample [1], while extensive gene content diversity within *Pelagibacter*, *Prochlorococcus,* and other predominant lineages of marine prokaryoplankton has been observed through prior cultivation-based and single-cell genomics studies [65–67]. The enduring difficulties in producing MAGs from *Pelagibacter*, *Prochlorococcus*, and other predominant genera of marine prokaryoplankton [57, 68, 69] imply that the degree of clonality in natural microbial populations may be lower than commonly assumed, likely due to extensive lateral gene transfer. This raises an important question of whether obtaining “complete” MAGs is the right goal in the representation of complex microbiomes. Since SAGs are produced from individual cells, population structure has no impact on their quality. Furthermore, the random nature of genome incompleteness in SAGs means that sequencing additional SAGs is compensatory, as demonstrated by the substantially better recovery of *Pelagibacter* and *Prochlorococcus* pangenomes by SAGs, as compared to MAGs, in this study (Fig. 3). Importantly, we show that even the largest current collections of surface ocean SAGs and MAGs are far from a complete pangenome representation, even for the most abundant microbial lineages in the ocean.

Genome assembly contamination with DNA sequences from other organisms, often called chimerism, is a serious quality concern and may lead to erroneous conclusions about an organism’s coding potential. In SAGs, chimerism may be caused by physical associations of multiple cells during cell sorting, reagent or instrument contamination with DNA, or errors in sequence read barcode demultiplexing. In MAGs, chimerism may be introduced by flaws in the algorithms for metagenome read assembly and binning. In agreement with several prior studies [5, 7, 51], we found a high frequency of chimerism (at the family level or higher) among OMD-M MAGs, while only one such chimera was detected among GORG-Tropics SAGs (Fig. 4). Due to the radically different methods in SAG and MAG generation, the definition of chimerism must also be viewed differently for these two methods. While a non-chimeric SAG is expected to represent DNA from a single cell, MAGs are by definition genomic consensuses of a multitude of related cells. It is generally assumed that genomic sequences aggregated in a MAG belong to the same microbial species, although this is not explicitly built into metagenome assembly and binning algorithms. Thus, improvements are needed in the biological definition of MAGs.

Our study emphasizes that the insufficient accuracy of current chimera detection methods can be a major challenge for scientists working with SAGs and MAGs. To a large extent, this is caused by the limited representation of the genomic content of complex microbiomes in the existing reference genome databases. Errors in chimera detection tools may be further exacerbated by the inclusion of chimeric genome assemblies in reference databases [7]. The integration of multiple approaches taken in this study improves confidence in genome contamination detection. However, the absence of true reference material constitutes an inherent challenge in the evaluation of SAG and MAG quality. An effective way to address these challenges is experimental validation of entire workflows of SAG and MAG generation using mock microbial communities, which should be comprised of strains with complete genome sequence information. This approach has been implemented in both SAG (including GORG-Tropics) [11, 61] and MAG [51, 64] studies, and we suggest that it should be applied more widely, particularly by laboratories engaged in large-scale SAG and MAG generation. An important added benefit of workflow validation with benchmark microorganisms is that it enables the analysis of quality metrics other than genome completeness and chimerism, such as the frequency of misassemblies, indels, and base mismatches [11], as well as potential biases in the recovery of various genome regions. Understanding these metrics is becoming increasingly important in the maturing field of genomics of uncultured microorganisms.

In summary, we found that randomized SAGs more accurately reflect the relative abundance and pangenome content of microbial lineages in the environment, and are less prone to chimerism than MAGs. SAGs are also better suited to link genome information with taxa discovered through 16S rRNA amplicon analyses. Meanwhile, MAGs have the advantage of more readily recovering genomes of the rare members of microbial communities that are statistically less likely to be represented among the same number of randomized SAGs. It is important to note that SAGs and MAGs can be produced using a wide range of laboratory and computational tools, and the quality of GORG-Tropics SAGs and OMD-M MAGs analyzed here does not fully represent the quality range of these two data types. However, GORG-Tropics and OMD-M are the first large collections of SAGs and MAGs, each consisting of thousands of genomes, obtained from the same environment, which offers a unique opportunity to assess their relative strengths and weaknesses. Therefore, we hope that the findings of this report will inform the design of research projects and data interpretation that involves SAGs or MAGs in future studies.

Although this report focuses exclusively on metrics of genome quality, a broader range of considerations is relevant to diverse users of SAGs and MAGs. For example, shotgun metagenome sequencing is a relatively inexpensive component of many environmental microbiology projects, and MAG generation from the obtained reads may not require a major additional effort. This makes MAG generation an attractive complement to many environmental microbiology projects, serving as a cost-effective tool contributing to the identification of the genomic context of genes of interest. SAG generation currently requires more complex and costly analytical procedures, making this approach less accessible to the broad research community for routine use. Due to reduced chimerism, improved recovery of non-clonal lineages, and more representative genome sampling, as compared to MAGs, SAGs are more suited for serving as references underlying microbiological cyberinfrastructure, such as tools for the recruitment and annotation of unassembled ‘omics fragments [1, 3, 70], genome taxonomic assignments [4] and genome quality control [5]. Given their sourcing from individual cells, SAGs offer several additional, unique opportunities, such as studies of microbial population genomics [67], direct matching of hosts and their mobile genetic elements and infections [55, 71, 72], and the integration of genome and phenome at single-cell resolution [73, 74]. Another important consideration is the continued, rapid improvement in metagenomic and single-cell genomic technologies, such as the recent breakthroughs in long-read sequencing [75] and droplet microfluidics [58, 76], which undoubtedly will improve the quality of both SAGs and MAGs and make both data types even more instrumental in environmental microbiology research.

### Supplementary Information

Additional file 1: Figure S1. Distribution of analyzed SAGs and MAGs by the depth (below sea level) from which their field samples were collected.

Additional file 2: Figure S2. Taxonomic representativeness at different ranks. Taxonomic composition revealed by randomized SAGs, MAGs, 16S rDNA amplicons (Amplicon) and shotgun metagenomic reads using mOTUs (Shotgun-M). Lineages that have ≥ 2% (or 3%) of the prokaryotic abundance estimated using either Amplicon or Shotgun-M, are shown in all the four method categories.

Additional file 3: Figure S3. Density distribution of GUNC’s clade separation score for the entire SAG and MAG datasets. Clade Separation Score (CSS) is binned at an interval of 0.01. The two major metrics generated by GUNC are used to estimate the degree of chimeric contamination for each dataset; the CSS is a measure of confidence when assigning a genome as a chimeric mixture, and genomes with CSSs larger than 0.45 (indicated by dashed lines) are considered as chimerism; the reference representation score (RRS) measures the closeness of a query genome represented by the reference in GUNC’s database; all genomes irrespective of their associated RRS are shown.

Additional file 4: Supplementary Table S1. Metadata of the analyzed SAG, MAG, and metagenomic samples.

Additional file 5: Supplementary Table S2. Metadata of the analyzed SAGs and MAGs.

Additional file 6: Supplementary Table S3. High-quality GenBank genomes for Pelagibacter and Prochlorococcus.

Additional file 7: Supplementary Table S4. A matrix showing the prevalence of KEGG-modules in Pelagibacter SAGs and MAGs.

Additional file 8: Supplementary Table S5. A matrix showing the prevalence of KEGG-modules in *Prochlorococcus* SAGs and MAGs.

Additional file 9: Supplementary Table S6. KEGG-modules prevalence found in KEGG-genomes of the Pelagibacter and Prochlorococcus.

Additional file 10: Supplementary Table S7. KEGG-modules that are determined to be present in Pelagibacter and Prochlorococcus based on prevalence criteria.

Additional file 11: Supplementary Table S8. The fraction of KOs shared by each pair of KEGG modules.

Additional file 12: Supplementary Table S9. Statistical test of standardized functional gene count difference between individual Pelagibacter SAGs and MAGs.

Additional file 13: Supplementary Table S10. Statistical test of standardized functional gene count difference between individual Prochlorococcus SAGs and MAGs.

Additional file 14: Supplementary Table S11. Estimated relative abundance of marine prokaryotic lineages using different methods.

Additional file 15: Supplementary Table S12. Statistical test of the estimated relative abundance difference between methods for each prokaryotic lineage.

Additional file 16: Supplementary Table S13. Statistical test of fragment recruitment rate difference between SAGs and MAGs using 24 metagenomic datasets.

Additional file 17: Supplementary Table S14. Key contamination related metrics provided by MDMcleaner and GUNC for all the analyzed SAGs and MAGs.

## Data Availability

GORG-Tropics SAGs and OMD MAGs were retrieved and downloaded under the BioProject accessions PRJEB33281 and PRJEB45951, respectively. The selected SAGs and MAGs for analyses in this study can be accessed at https://figshare.com/articles/dataset/SAG-MAG_comparison_analyzed_genomes/23949138. The accession numbers of the analyzed metagenomic reads and assemblies were provided in Supplementary Table S1.
