# Supplementary figures and images for: Genomic representativeness and chimerism in large collections of SAGs and MAGs of marine prokaryoplankton
Source: Microbiome. 2024 Jul 15;12:126. doi: 10.1186/s40168-024-01848-3 (PMC11247762; doi:10.1186/s40168-024-01848-3)

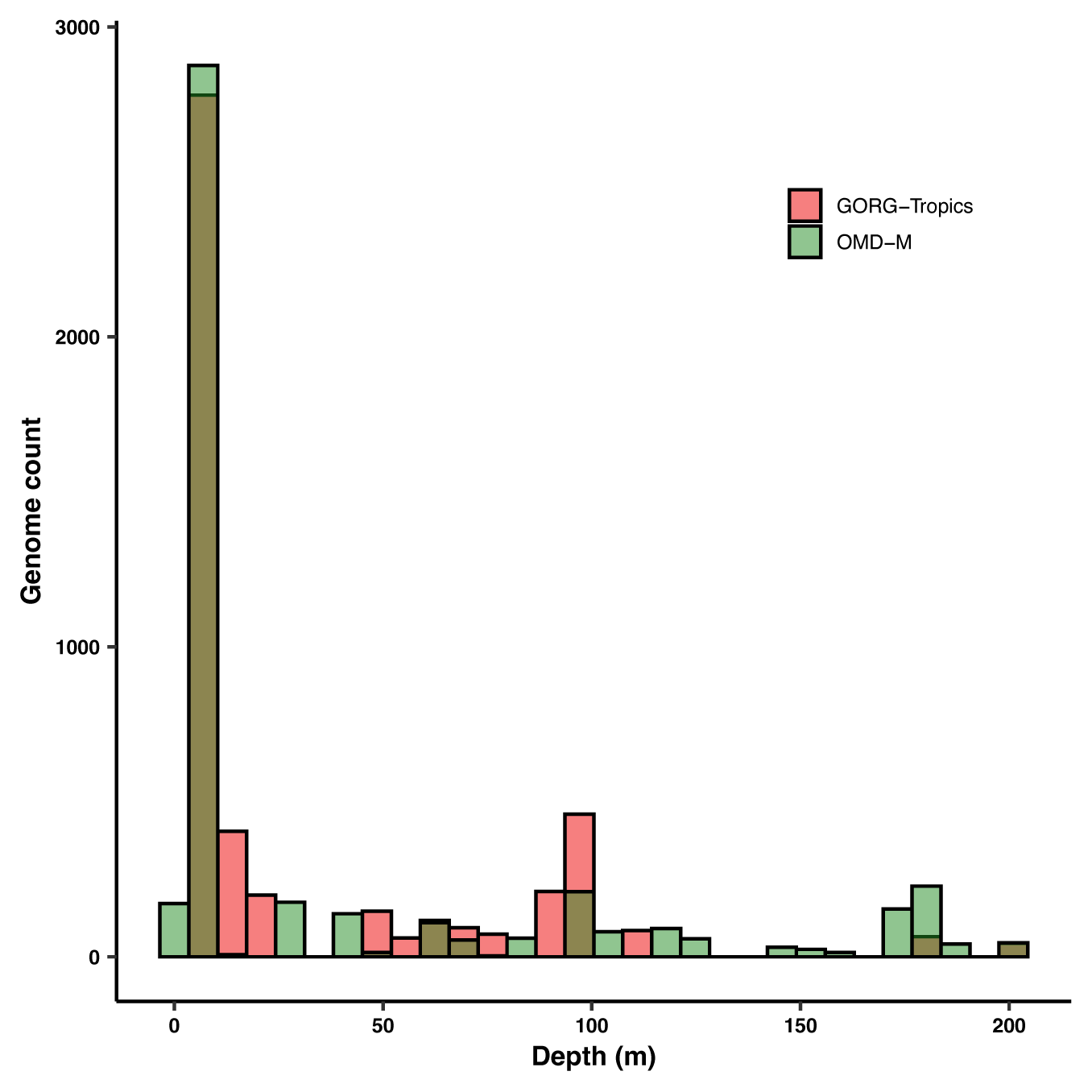

Supplement: Supplementary file 2 — Additional file 1: Figure S1. Distribution of analyzed SAGs and MAGs by the depth (below sea level) from which their field samples were collected. [file 40168_2024_1848_MOESM1_ESM.tif]

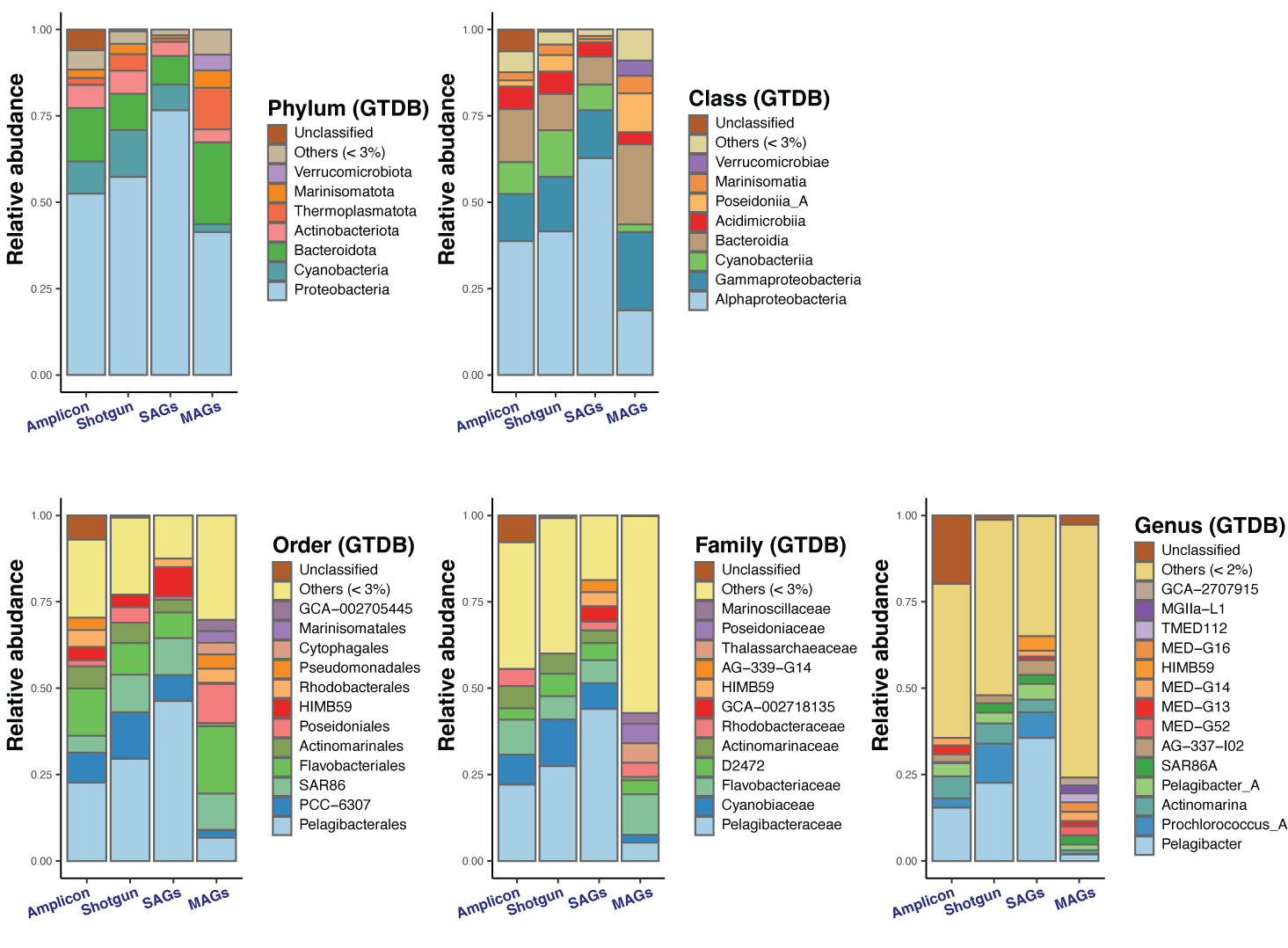

Supplement: Supplementary file 3 — Additional file 2: Figure S2. Taxonomic representativeness at different ranks. Taxonomic composition revealed by randomized SAGs, MAGs, 16S rDNA amplicons (Amplicon) and shotgun metagenomic reads using mOTUs (Shotgun-M). Lineages that have ≥ 2% (or 3%) of the prokaryotic abundance estimated using either Amplicon or Shotgun-M, are shown in all the four method categories. [file 40168_2024_1848_MOESM2_ESM.tif]

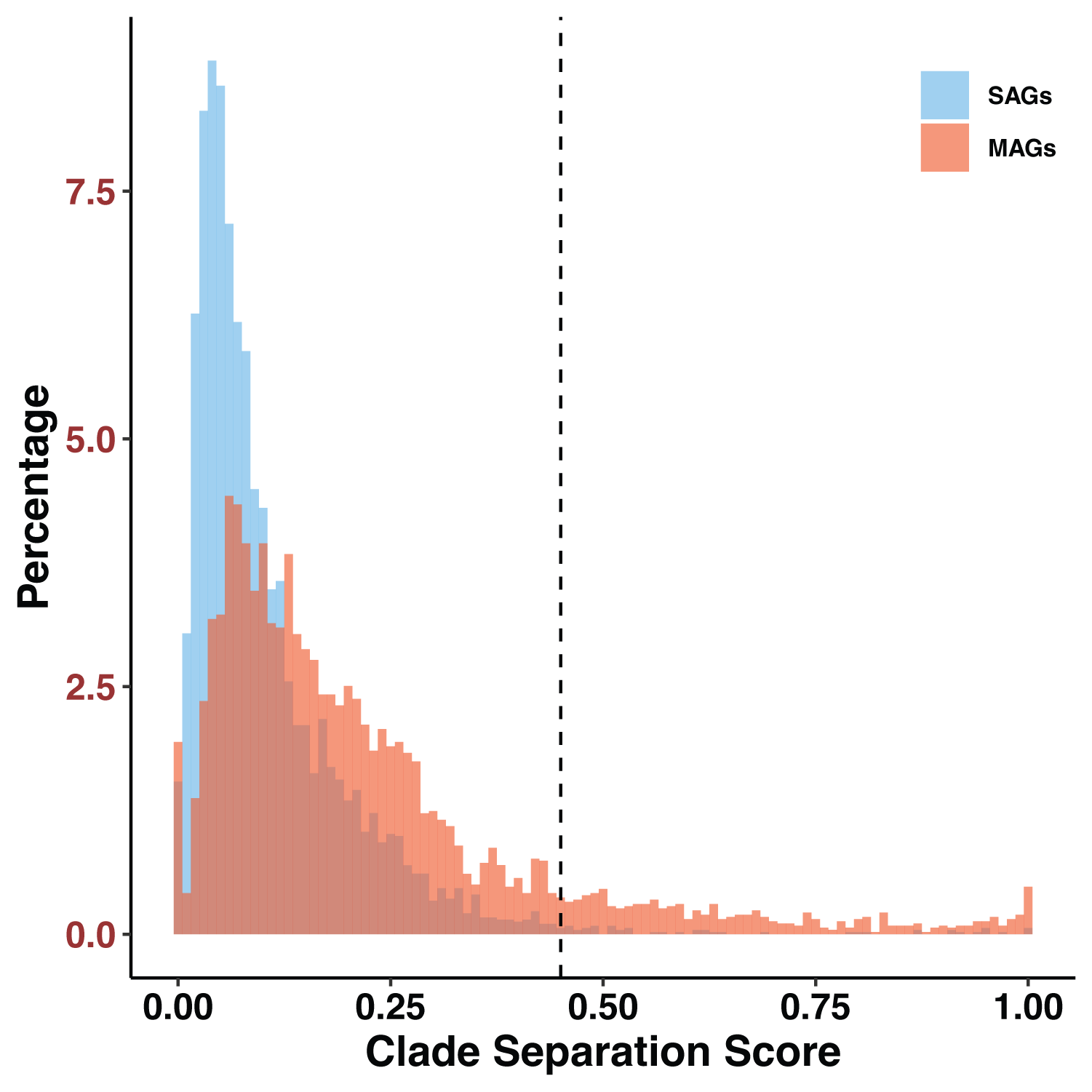

Supplement: Supplementary file 4 — Additional file 3: Figure S3. Density distribution of GUNC’s clade separation score for the entire SAG and MAG datasets. Clade Separation Score (CSS) is binned at an interval of 0.01. The two major metrics generated by GUNC are used to estimate the degree of chimeric contamination for each dataset; the CSS is a measure of confidence when assigning a genome as a chimeric mixture, and genomes with CSSs larger than 0.45 (indicated by dashed lines) are considered as chimerism; the reference representation score (RRS) measures the closeness of a query genome represented by the reference in GUNC’s database; all genomes irrespective of their associated RRS are shown. [file 40168_2024_1848_MOESM3_ESM.tif]
